# The influence of social support on sleep and fatigue level among patients receiving hemodialysis in Saudi Arabia: a cross-sectional correlational design

**DOI:** 10.3389/fpsyg.2023.1272500

**Published:** 2023-12-12

**Authors:** Bushra Alshammari, Sameer A. Alkubati, Eddieson Pasay-an, Awatif Alrasheeday, Norah Madkhali, J. Silvia Edison, Venkat Bakthavatchaalam, Marim Saud Alshammari, Amnah Ayed AlRashidi, Farhan Alshammari

**Affiliations:** ^1^Department of Medical Surgical Nursing, College of Nursing, University of Hail, Hail, Saudi Arabia; ^2^Department of Nursing, Faculty of Medicine and Health Sciences, Hodeida University, Hodeida, Yemen; ^3^Department of Nursing Administration, College of Nursing, University of Hail, Hail, Saudi Arabia; ^4^Department of Nursing, College of Nursing, Jazan University, Jazan, Saudi Arabia; ^5^School of Engineering, Cardiff University, Cardiff, Wales, United Kingdom; ^6^Department of Health Awareness, King Salman Specialist Hospital, Hail Health Cluster, Hail, Saudi Arabia; ^7^Department of Obstetrics and Gynecology, Hail General Hospital, Hail Health Cluster, Hail, Saudi Arabia; ^8^Department of Pharmaceutics, College of Pharmacy, University of Hail, Hail, Saudi Arabia

**Keywords:** social support, fatigue, sleep quality, hemodialysis, Saudi Arabia

## Abstract

**Background:**

Patients on hemodialysis (HD) are more likely to experience sleep problems and fatigue that may affect their health outcomes. Management of these patients with social support may improve their sleep quality and fatigue as well as their health.

**Aim:**

This study aimed to assess the influence of social support on sleep quality and fatigue levels among HD patients.

**Methods:**

A cross-correlational study was conducted among 260 conveniently sampled HD patients from four dialysis centers in Hail and Al-Qassim cities of Saudi Arabia from Jun 2022 to January 2023. Besides sociodemographic data, the Pittsburgh Sleep Quality Index (PSQI), the Multidimensional Assessment of Fatigue (MAF) and the Oslo Social Support Scale (OSSS-3) were used to assess sleep quality, fatigue levels and social support, respectively. Chi-square test was used to determine the association between categorical variables, while Pearson’s correlation coefficient was used to test the correlation between sleep quality, fatigue, and social support.

**Results:**

Poor sleep and high fatigue were significantly higher in older patients compared to younger patients (*p* <0.001), while strong social support was significantly lower in older patients than younger and middle-aged ones (*p* = 0.001). On the other hand, poor sleep and high fatigue were significantly higher in males than females (*p* = 0.022 and *p* <0.001, respectively), while strong social support was significantly higher in females than males (*p* <0.001). Married patients showed significantly poorer sleep than single ones (*p* = 0.019), but single patients received significantly stronger social support. Retired patients showed significantly poorer sleep, higher fatigue and weaker social support than other groups (*p* <0.001). There was a significant negative correlation between fatigue and sleep quality among HD patients, where patients with more fatigue had poorer sleep (*r* = −0.510, *p* <0.001). A significant positive correlation was found between social support and sleep quality, where patients with stronger social support had more normal sleep (*r* = 0.415, *p* <0.001). However, a significant negative correlation was found between social support and fatigue, where patients with stronger social support had lower levels of fatigue (*r* = −0.479, *p* <0.001).

**Conclusion:**

Saudi patients on HD who have stronger social support have better sleep quality and reduced fatigue levels than those with less social support. There is a need to design and implement intervention studies with structured social support programs, and to evaluate their effectiveness on improving sleep and reducing fatigue among HD patients.

## 1 Introduction

Between 1990 and 2017, the global prevalence of chronic kidney disease (CKD) increased across all age groups by 29.3% (95% uncertainty interval [UI]: 26.4 to 32.6) while remaining consistent in all age groups (1.2, 95% UI: 1.1 to 3.5) (Collaboration GBDCKD, 2020). Saudi Arabia is no exception because the country’s age-standardized prevalence of CKD (stages 1–5, excluding renal replacement therapy) is higher (5,446 per 100,000) than that in Western Europe and North America (Alsuwaida et al., 2010). Due to poor quality of life (QOL) associated with higher rates of hospitalization, hemodialysis (HD), and mortality, the rising global trend of CKD represents a health risk. For instance, patients on HD, particularly the elderly, are more likely to experience sleep problems that can lead to chronic fatigue syndrome (Lindner et al., 2015) and uremia-related metabolic disorders (Maung et al., 2016), both of which have a negative impact on the QOL. As a result, HD has tremendous repercussions related to patients’ daily lives, including social, economic, and physical effects (such as fatigue syndrome), significantly affecting both patients and their support network.

Sleep disorders and fatigue are notably prevalent in patients undergoing HD (Al Naamani et al., 2021) Studies have shown that a significant proportion of HD patients experience sleep-related problems, which include insomnia, restless leg syndrome, and sleep apnea. The prevalence rates of sleep disturbances among HD patients, as revealed by several studies worldwide, range from 40 to 85% (Al Naamani et al., 2021; Alshammari et al., 2023) Several factors contribute to the high prevalence of sleep disorders in HD patients. Factors such as stress, dietary restrictions, limited physical activity, and the dialysis schedule can interfere with normal sleep routines and contribute to a sense of fatigue (Hsouna et al., 2022; Abdul Rahim et al., 2023; Taheri et al., 2023) Poor sleep and fatigue have been linked to worse clinical outcomes in HD patients, including higher morbidity and mortality rates (Kumar et al., 2010) They are associated with an increased risk of cardiovascular events, which are a leading cause of death in this population (Zhang et al., 2014).

Patients on HD exhibit limited functional capacity, generalized muscle weakness, and reduced levels of physical activity, all of which contribute to fatigue (Zyga et al., 2015). According to Bossola et al. (2017), fatigue is one of the most frequent complaints of HD, which considerably lowers patients’ QOL and is linked to both cardiac-related and all-cause deaths (Bossola et al., 2017). Fatigue has been reported to be prevalent among 42–97% of HD patients (Horigan, 2012; Sakkas and Karatzaferi, 2012; Burdelis and Cruz, 2023). Nevertheless, this serious and life-threatening health condition in the HD population is often ignored and left untreated (Zyga et al., 2015; Debnath et al., 2021), being a strong predictor of poor health-related QOL and poor survival (van der Borg et al., 2021). Moreover, fatigue can be associated with anemia, mood disorders, and malnutrition (Debnath et al., 2021).

The impact of social support on the QOL of HD patients has been argued to warrant research (Pan et al., 2019; Alshraifeen et al., 2020) because such support can encourage HD patients to adopt more positive attitudes toward their condition and general well-being (Alshraifeen et al., 2020) and may improve health outcomes (Theodoritsi et al., 2016). Social support early in HD is linked to survival and well-being, considerably improving patients’ outcomes. The level of social support is crucial for better adaptation to the chronic nature of the illness, sleep problems, and likely outcomes during HD (Pan et al., 2019). Social support is directly correlated with increased QOL, better acclimatization to HD, and adherence to treatment regimens recommended by physicians and caretakers (Kim et al., 2018; Alshraifeen et al., 2020) Therefore, healthcare professionals and social service providers can focus on and strengthen social support networks of HD patients as a crucial aspect of care if they become aware of the impact of perceived social support on their sleep quality (Mohamed et al., 2023).

Early studies found a link between social support and fatigue (Karadag et al., 2013), social support and sleep quality (Stafford et al., 2017; Mohamed et al., 2023). Among the myriad complications associated with CKD, patients undergoing HD frequently experience sleep disorders and fatigue, leading to a marked reduction in their quality of life. While these issues have been extensively documented, the specific role of social support in mitigating these challenges remains underexplored, especially in the Saudi Arabian context. Additionally, the complex interaction between social support, sleep quality, and fatigue levels in HD patients is an area that has not been thoroughly investigated. Understanding the role of social support can have significant implications for the treatment and management strategies for HD patients. This study aims to fill this gap by assessing the impact of social support on sleep quality and fatigue among HD patients in Saudi Arabia. We hypothesize that robust social support networks will correlate with improved sleep quality and reduced fatigue, thereby enhancing patient outcomes.”

## 2 Materials and methods

### 2.1 Study design and setting

This cross-sectional correlational design was conducted in four dialysis centers in Hail and Al-Qassim cities of Saudi Arabia.

### 2.2 Sample and sampling technique

This study enrolled 260 conveniently sampled HD patients from Jun 2022 to January 2023. The rationale for employing convenient sampling in this study was primarily driven by practical considerations. These included accessibility to the patient population, resource constraints, and the specific context of the study, which necessitated a more readily available sample. Convenient sampling allowed us to efficiently gather data from a specific subgroup of the population who were readily accessible and willing to participate. This was particularly crucial given the challenges associated with engaging HD patients, who often face significant health and logistical burdens that can impede participation in research studies. The study included patients with chronic renal failure who had been undergoing HD for more than three months and were willing to participate. Patients were excluded from the study if they were under 18 years of age, did not consent to participate, or had cognitive disorders or severe complications.

Nursing staff at the HD centers, serving as gatekeepers, facilitated access to the study environments and participants. These gatekeepers took charge of initially screening potential participants to assess their interest in joining the study. Eligible patients were then referred to the researcher by these gatekeepers. The researcher subsequently provided these patients with an invitation letter, an information sheet, and a consent form. Patients had a 48-h period to express their willingness to participate in the study. Following their agreement, the questionnaires were distributed for them to complete.

### 2.3 Data collection tools

Besides the questionnaire used to collect socio-demographic data such as age, gender, marital status, occupation, and level of education, data were collected using three tools. The first tool was the Pittsburgh Sleep Quality Index (PSQI), which was used to assess sleep quality in HD patients (Buysse et al., 1989). Subjective sleep quality, sleep latency, sleep length, habitual sleep efficiency, sleep disruptions, use of sleep medicines, and daytime dysfunction were the seven components of this tool. It consisted of 19 questions with a possible score range of 0 to 21. A lower score denotes average sleep quality, while a higher score denotes poor sleep quality. Patients with a PSQI global score of 5 or more were considered to sleep poorly, while those with a score of less than 5 were considered to sleep normally (Buysse et al., 1989; Amare et al., 2022).

The second tool was the Arabic version of the Multidimensional Assessment of Fatigue (MAF), which was used to assess the level of fatigue among HD patients. It included 16 items to measure four domains of fatigue: severity, discomfort, timing, and effect on daily activities. The first 14 items were scored on a scale of 1 (Not at all) to 10 (A great deal), while the last two items were scored on a 4-point Likert scale. The possible MAF scores ranged from 1 (no fatigue) to 50 (severe fatigue). Fatigue was divided into two categories: low fatigue (scores 1 to 25) and high fatigue (scores 26 to 50). Permission to use the Arabic version of MAF was granted by the Mapi Research Trust. The tool’s validity and reliability have been reported in a variety of languages, diseases, and settings across the world (Belza et al., 2018).

The third tool was the Oslo Social Support Scale (OSSS-3), which was used to assess the level of social support (Kocalevent et al., 2018). It included three questions: “*How many people are so close to you that you can count on them if you have great personal problems?*,” “*How much interest and concern do people show in what you do?*,” and “*How easy is it to get practical help from neighbors if you should need it?*.” The total OSSS-3 score ranged from 3 to 14, with high scores signifying great levels of social support and low scores signifying weak levels. Social support was then divided into three categories: poor (scores 3 to 8); moderate (scores 9 to 11) and strong (scores 12 to 14) (Kocalevent et al., 2018; Zhu et al., 2023). The researchers subjected the three tools for the cultural adaptation and validation in the context of Saudi Arabian HD patients which includes translation of the items from English to Arabic, review and refinement, and pilot testing for 20 patients who were not involved in the study. The results of the reliability of the PSQI, MAF, and OSSS-3 questionnaires were α = 0.724, 0.883, 0.925, respectively The adaptation and validation results suggest that the tools were reliable, valid, and sensitive for establishing the measures of the three tools. Further, the researchers compared the cultural equivalence of the tools from the data gathered from the HD patients in Saudi Arabia with the data from other populations. Hence, it can be concluded that PSQI, MAF, and OSSS-3 are culturally appropriate and valid comparable with the original version. Such a result can be used to help in measuring support services and interventions for HD patients in Saudi Arabia.

### 2.4 Ethical considerations

This study was approved by the Research Ethics Committees at the University of Hail, Hail Health Clusters and the General Directorate of Health Affairs in Al-Qassim (Approval No.: H-2021-206, H-08-L-074, and 607-44-2091, respectively). Anonymity and confidentiality were maintained during the study.

### 2.5 Data analysis

Data were analyzed using the IBM SPSS Statistics software, Version 27 (IBM Corp., Armonk, NY, United States). Mean and standard deviation (SD) were used to describe normally distributed continuous variables, whereas frequencies and percentages were used to describe categorical variables. Chi-square test was used to determine the association between categorical variables, while Pearson’s correlation coefficient was used to test the correlation between sleep quality, fatigue, and social support. Statistical significance was set at *p* < 0.05.

## 3 Results

Table 1 shows that age and gender of HD patients were significantly associated with sleep quality, fatigue and social support. Poor sleep and high fatigue were significantly higher in older patients compared to younger patients (*p* <0.001), while strong social support was significantly lower in older patients than younger and middle-aged ones (*p* = 0.001). On the other hand, poor sleep and high fatigue were significantly higher in males than females (*p* = 0.022 and *p* <0.001, respectively), while strong social support was significantly higher in females than males (*p* <0.001). Married patients showed significantly poorer sleep than single ones (*p* = 0.019), but single patients received significantly stronger social support (*p* <0.001). However, there was no significant difference in fatigue level by marital status (*p* = 0.212). Retired patients showed significantly poorer sleep, higher fatigue and weaker social support than other groups (*p* <0.001). On the other hand, patients’ level of education was not significantly associated with sleep quality, fatigue, or social support.

**Table 1:** Association of sociodemographic characteristics of HD patients with their sleep quality, fatigue and social support levels in Hail and Al-Qassim cities, Saudi Arabia (2022–2023).

Variable	*N*		Sleep quality *n* (%)		Fatigue n (%)		Social support *n* (%)		
			Normal	Poor	Low	High	Mid	Moderate	Strong
Age (years)	<35	54	39 (72.2)	15 (27.8)	37 (68.5)	17 (31.5)	5 (9.3)	27 (50.0)	22 (40.7)
	35–50	90	69 (76.7)	21 (23.3)	53 (58.9)	37 (41.1)	10 (11.1)	48 (53.3)	32 (35.6)
	>50	116	54 (46.6)	62 (53.4)	44 (37.9)	72 (62.1)	29 (25.0)	67 (57.8)	20 (17.2)
			***p* <0.001**		***p* <0.001**		***p* = 0.001**		
Gender	Male	149	84 (56.4)	65 (43.6)	59 (39.6)	90 (60.4)	37 (24.8)	84 (56.4)	28 (18.8)
	Female	111	78 (70.3)	33 (29.7)	75 (67.6)	36 (32.4)	7 (6.3)	58 (52.3)	46 (41.4)
			***p* = 0.022**		***p* <0.001**		***p* <0.001**		
Marital status	Married	215	127 (59.1)	88 (40.9)	107 (49.8)	108 (50.2)	40 (18.6)	124 (57.7)	51 (23.7)
	Single	45	35 (77.8)	10 (22.2)	27 (60.0)	18 (40.0)	4 (8.9)	18 (40.0)	23 (51.1)
			***p* = 0.019**		***p* = 0.212**		***p* <0.001**		
Occupational status	Employee	75	46 (61.3)	29 (38.7)	39 (52.0)	36 (48.0)	14 (18.7)	43 (57.3)	18 (24.0)
	Unemployed	102	71 (69.6)	31 (30.4)	64 (62.7)	38 (37.3)	6 (5.9)	59 (57.8)	37 (36.3)
	Student	15	13 (86.7)	2 (13.3)	14 (93.3)	1 (6.7)	1 (6.7)	5 (33.3)	9 (60.0)
	Retired	68	32 (47.1)	36 (52.9)	17 (25.0)	51 (75.0)	23 (33.8)	35 (51.5)	10 (14.7)
			***p* = 0.005**		***p* <0.001**		***p* <0.001**		
Level of education	Illiterate	61	37 (60.7)	24 (39.3)	34 (55.7)	27 (44.3)	10 (16.4)	34 (55.7)	17 (27.9)
	Primary education	32	19 (59.4)	13 (40.6)	18 (56.3)	14 (43.8)	5 (15.6)	18 (56.3)	9 (28.1)
	Secondary education	115	73 (63.5)	42 (36.5)	55 (47.8)	60 (52.2)	24 (20.9)	62 (53.9)	29 (25.2)
	Higher education	52	33 (63.5)	19 (36.5)	27 (51.9)	25 (48.1)	5 (9.6)	28 (53.8)	19 (36.5)
			***p* = 0.963**		***p* = 0.717**		***p* = 0.621**		

Table 2 shows that the level of social support was significantly associated with the quality of sleep and fatigue level (*p* <0.001). Patients who received strong social support had normal sleep, while patients receiving mild social support had poor sleep. On the other hand, patients who received strong social support had significantly lower fatigue levels compared to those receiving less social support (*p* < 0.001).

**Table 2:** Impact of social support on sleep quality and fatigue among HD patients in Hail and Al-Qassim cities, Saudi Arabia.

Variables		*N*	Social support category *n* (%)			*p*-value
			Mild	Moderate	Strong	
Sleep quality	Normal	162	7 (15.9)	81(57.0)	74 (100)	<0.001
	Poor	98	37 (84.1)	61(43.0)	0 (0.0)	
Fatigue	Low	134	10 (22.7)	63 (44.4)	61 (82.4)	<0.001
	High	126	34 (77.3)	79 (55.6)	13 (17.6)	

**Figure 1:**
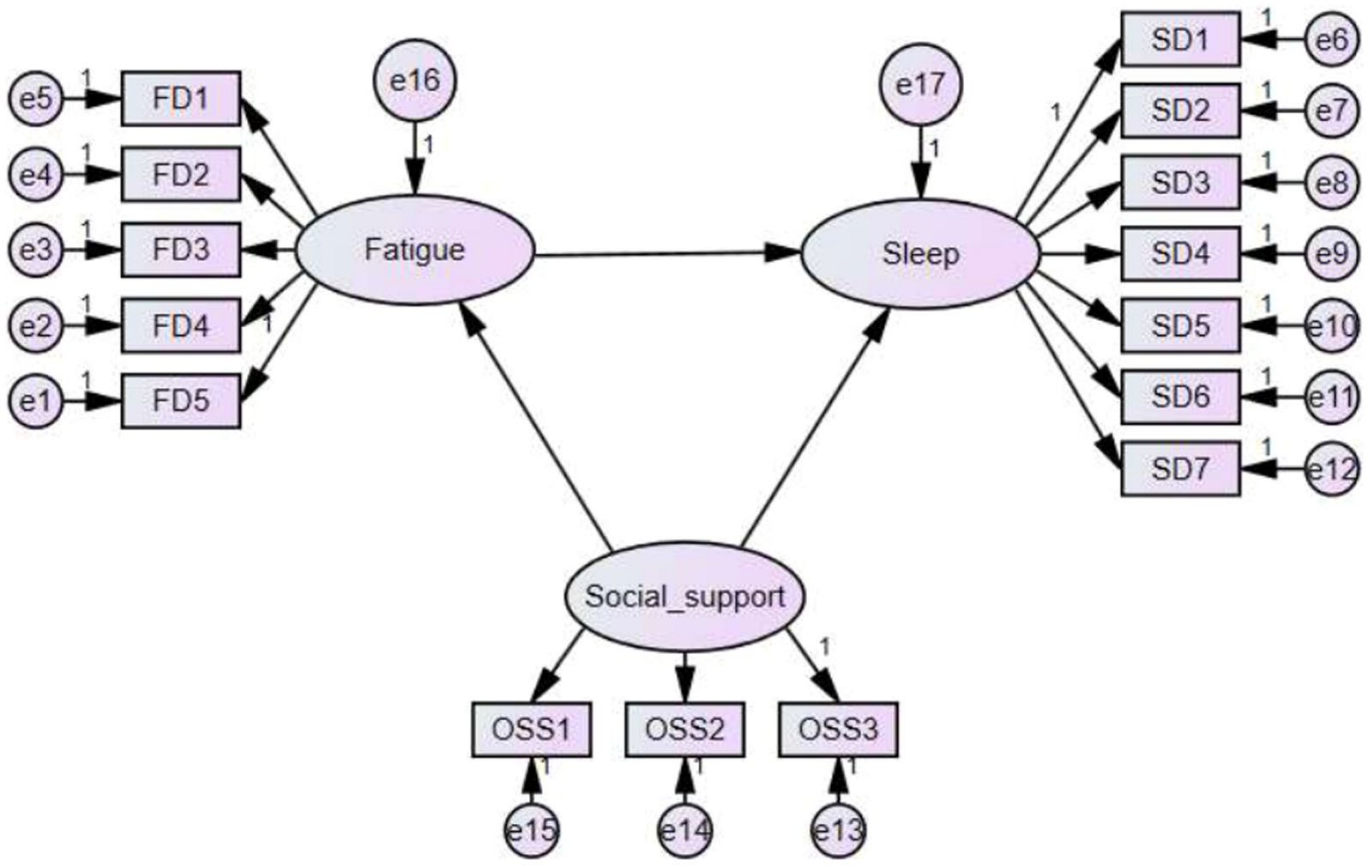
Emerging model of the impact of social support on sleep quality and fatigue among HD patients. Abbreviations: FD, fatigue domain; SD, sleep domain; OSS, Oslo Social Support.

Table 3 shows a significant negative correlation between fatigue and sleep quality among HD patients, where patients with more fatigue had poorer sleep (*r* = −0.510, *p* <0.001). A significant positive correlation was found between social support and sleep quality, where patients with stronger social support had more normal sleep (*r* = 0.415, *p* <0.001). However, a significant negative correlation was found between social support and fatigue, where patients with stronger social support had lower levels of fatigue (*r* = −0.479, *p* <0.001; Figure 1).

**Table 3:** Correlation between social support, sleep quality and fatigue levels among HD patients in Hail and Al-Qassim cities, Saudi Arabia.

Variable			Estimate	SE	*r*	*p*-value
Fatigue	←	Social support	−0.479	0.092	−5.725	<0.001
Sleep	←	Fatigue	−0.510	0.055	−3.912	<0.001
Sleep	←	Social support	0.415	0.070	6.785	<0.001

SE, standard error; *r*, Pearson’s correlation coefficient, MSA.

## 4 Discussion

To the best of the researchers’ knowledge, this is the first study to investigate the impact of social support on sleep quality and fatigue level among HD patients in Saudi Arabia. Determining sleep quality and its impact on QOL and other health-related outcomes in HD patients can help to develop or enhance professional practices for strengthening patients’ social support and networks. This study revealed a significant association between the age of patients and sleep quality, fatigue, and social support, with older patients having significantly poorer sleep and fatigue than younger ones. This finding suggests a probable link between advancing age and a greater likelihood of developing sleep problems and fatigue among HD patients. Poor sleep and extreme fatigue are more common in elderly HD patients for several reasons, including age-related changes in sleep patterns and architecture. Typically, older adults have more fragmented sleep, less profound sleep, and a greater tendency to awaken throughout the night (Firoz et al., 2016). These age-related alterations can exacerbate sleep difficulties in older HD patients. In line with this study, poor sleep quality and reduced sleep efficiency were both identified in elderly patients on peritoneal dialysis (Zhang et al., 2021).

In addition to renal failure, elderly patients frequently suffer from diabetes, cardiovascular disease, and arthritis (Jaul and Barron, 2017). As a result of these health conditions, financial concerns, or social isolation, seniors may experience increased psychological stress (Miner and Kryger, 2017), compromising their sleep quality. The HD procedure can be physically stressful and unpleasant, particularly for elderly patients with age-related limitations (Wang et al., 2016). Extended periods of sitting during HD, potential discomfort at the site of needle insertion, and the need to limit fluid consumption can all affect sleep quality and contribute to fatigue (Fonseca et al., 2016).

In this study, approximately half of patients across all age groups received moderate social support. Yet, older patients received significantly weaker social support than younger and middle-aged patients. This finding implies that older patients may experience greater social isolation and diminished access to various forms of assistance and emotional support, leading to several repercussions such as a decline in psychological health, a lack of practical assistance, a decline in QOL, and an increase in the healthcare burden. When compared to younger patients, those undergoing HD in their later years are more likely to suffer negative consequences. Complications, including hemodynamic instability and depression, occur more frequently and contribute to a greater mortality rate (Ahmed and Catic, 2018). When coupled to conservative therapy, HD is associated with a significant increase in the treatment burden for elderly patients (van Oevelen et al., 2021). Nurses can provide comprehensive palliative or supportive care not involving HD that can be potentially effective as a therapeutic option for certain elderly people (Raj et al., 2020). Generally, the reduced levels of social support among older HD patients can negatively impact their physical and emotional health, treatment adherence, and overall QOL. Therefore, it is essential to recognize and resolve these disparities in order to provide comprehensive care to this vulnerable population.

In this study, male patients showed significantly poorer sleep and higher fatigue levels than females with weaker social support, implying that HD patients have drastically different sleep habits based on gender. Sleep apnea may cause more sleep disruption in males than in females (Snyder and Cunningham, 2018). Males typically have lower levels of melatonin than females (Ruge et al., 2019). Melatonin is a hormone that is involved in the control and regulation of sleep. However, this finding contradicts that reported in other studies. (Ruge et al., 2019). For instance, a significant proportion of Iranian patients on HD, regardless of gender, were found to have trouble sleeping (Sabet et al., 2012). On the other hand, a review study also revealed that women may have higher fatigue levels than men (Horigan, 2012). In Turkey, more than two-thirds of HD patients reported receiving social support from their families, the gender differences between the levels of social support received by patients were not compared (Baskan et al., 2020). There are several facets to the concept of poor sleep, fatigue, and lack of social support. This concept is influenced by a variety of variables and can manifest differently in different people, independent of gender. To get conclusive findings, additional studies are required to compare the degrees of insufficient sleep, fatigue, and social support between both genders in the context of HD.

Marital status was found to be significantly associated with sleep quality, but not with fatigue level, among patients in the present study, where married patients had significantly poorer sleep than single patients. This finding indicates that fatigue can be affected by different factors such as physical exertion, mental tension, medical conditions, and lifestyle choices. The finding of the present study is consistent with that reported by Firoz et al., who found that sleep quality of married HD patients in Iran was much lower than that of their single counterparts (Norozi Firoz et al., 2019). Married patients may find it more difficult to get adequate rest due to additional responsibilities at home (Eldridge-Smith et al., 2023). They may become more stressed due to concerns for their health and the well-being of their families (Brown et al., 2019), making it more difficult to get adequate sleep. Furthermore, patients on HD in Greece were found not to differ from the general population in terms of fatigue levels based on marital status (Tsirigotis et al., 2022). Given the potential impact of marital relationships on sleep quality, nurses should engage patient’s family members or spouse in any discussions about treating sleep and fatigue. Nurses can educate the patient’s family members on strategies to help the patient sleep, such as providing an environment that is conducive to sleep and encouraging a regular sleep routine.

The significantly stronger social support received by single patients compared to married ones implies that there may be specific dynamics or elements behind this support. For instance, being single may allow for greater flexibility in terms of time and availability to participate in social activities, seek support, and build better social networks (Apostolou and Esposito, 2020; Park and MacDonald, 2022; Park et al., 2023). Married people may have additional responsibilities and obligations within their marriage and family, limiting their ability to provide or receive social support (van Oevelen et al., 2021). Some married HD patients, however, may have strong social support networks, and some single patients may have limited support (Kukihara et al., 2020; Unsal Avdal et al., 2020). Nurses must account for individual differences when exploring the nature of social support needed for HD patients.

This study revealed poorer sleep and higher fatigue levels with weaker social support in retired patients compared to their counterparts. This finding shows that retirement can cause alterations in sleep and energy patterns. In Sweden, fatigue was found to decrease in all age groups over eight years, while sleep problems increased (Åkerstedt et al., 2018). In addition, a cross-sectional survey in Northern California revealed that 9% of elderly adults experienced frequent daytime fatigue, regardless of gender, and that adults aged 70–74 years were less likely to have fatigue than younger and older age groups (Gordon et al., 2022). Retired patients typically have less social support than other groups (Myllyntausta et al., 2021). For instance, sleep-related impairment in physicians was found to be associated with diminished professional satisfaction, which may be due to a lack of social support (Trockel et al., 2020). Therefore, retired patients may be at risk for poor sleep, fatigue, and social isolation, which can have a negative impact on their health and well-being.

The present study found no significant association between educational levels and sleep quality, fatigue level, and social support. In this context, educational attainment was found to have no direct influence on these factors in the HD patient population (Parvan et al., 2013). In HD patients, other factors such as depression, perceived social support, and the presence of other psychological disorders were found to be more closely associated with sleep quality and overall well-being (Firoz et al., 2016; Mohamed et al., 2023).

In the present study, social support level significantly affected sleep quality, showing that social support may play a role in the quality of sleep among HD patients. Patients who received stronger social support had normal sleep, while patients who received weak social support had poorer sleep. Numerous studies on the connection between social support and sleep quality of HD patients found that a lack of social support can be a significant predictor of poor sleep quality (Pan et al., 2019; Mohamed et al., 2023). In particular, a high level of perceived support from one’s family and friends were found to be a strong predictor of poor sleep quality (Mohamed et al., 2023). Other factors that have been found to alter the sleep quality of HD patients include depression, weariness, and increased serum phosphate levels (Ng et al., 2020; Ho et al., 2022). On the other hand, it was found that social support mediates the relationship between sleep problems, depression, and health-related QOL in HD patients (Pan et al., 2019). To address sleep problems in HD patients, nurses should account for patients’ social support systems because social support can impact their overall well-being and health.

The finding that strong social support led to lower fatigue levels in the present study shows the significant role that social support plays in managing fatigue in HD patients. Likewise, a negative correlation was reported between fatigue severity among Turkish HD and the levels of social support from friends, family, and significant others (Karadag et al., 2013). A high level of social support was correlated with less fatigue, and patients with high levels of support from family, friends, a particular person, and overall had lower mean scores on all of these dimensions (Karadag et al., 2013). In Makkah, Saudi Arabia, social support was found to be significant in helping HD patients to combat fatigue (Garwai et al., 2020). Fatigue is a common side effect of HD that can negatively impact patients’ QOL (Tsirigotis et al., 2022). The need for social support in HD patients is determined by factors such as the size and quality of their social network, as well as the severity of their condition (Asiri et al., 2023).

The present study revealed a negative correlation between fatigue and sleep quality, with patients experiencing higher fatigue showed poorer sleep quality. This finding is consistent with that reported in other studies (Joshwa et al., 2012; Al Naamani et al., 2021). A direct correlation was found between sleep deprivation and feelings of fatigue among Indian HD patients (Joshwa et al., 2012). Poor sleep quality was also found to be more frequent among Iranian HD patients on maintenance dialysis, with mood disorders and HD being its significant predictors (Masoumi et al., 2013). A study on HD patients during the COVID-19 pandemic showed a correlation between fatigue and poor sleep quality (Percze et al., 2023). These findings add to the growing body of evidence that HD patients frequently struggle with poor sleep quality.

In addition, a positive correlation was found between social support and sleep quality among HD patients in the present study, with patients receiving strong social support sleeping more normally. This finding suggests that the existence of strong social support is associated with a higher likelihood of having normal sleep patterns among HD patients. A recent studies found that perceived stress was positively correlated with insomnia, while social support was negatively correlated with insomnia (Mohamed et al., 2023; Tao et al., 2023). Furthermore, poor sleep quality was significantly correlated with both the perception of support from friends and the perception of total social support (Mohamed et al., 2023). In Malaysia, HD patients receiving social support were more likely to survive (Ng et al., 2020). As part of an all-encompassing strategy for the management of sleep disorders in the HD population, nurses should consider integrating techniques that strengthen social support.

The present study also found a negative correlation between social support and fatigue, with patients who had strong social support showing lower levels of fatigue. Similarly, a negative correlation was established between the degree of exhaustion and the presence of social support from family and friends among Turkish HD patients (Karadag et al., 2013), where patients who suffered from extreme exhaustion also reported lower levels of support from their social networks on average. Another study concluded that social support has a direct association with increased treatment compliance, adaptation to dialysis treatments, and QOL of Jordanian HD patients (Alshraifeen et al., 2020). This supports the idea that increasing the levels of social support can lead to better overall well-being as well as disease management.

The study has several limitations: The study utilized a convenience sampling method, which poses a risk of bias and may affect the representativeness of the population studied. Additionally, gatekeepers’ involvement could restrict access to specific patient groups. The study also employed a cross-sectional and quantitative methodology. For a deeper exploration of the phenomenon, a longitudinal and qualitative approach is suggested. The study also solely focus on HD patients in Saudi Arabia, future research should consider including a more diverse and geographically varied sample. This broader approach would enhance the generalizability of the findings to other populations. Additionally, conducting comparative studies across different countries or cultural contexts could provide valuable insights into how regional and cultural factors influence the experiences of HD patients.

## 5 Conclusion

Saudi patients on HD who have stronger social support have better sleep quality and reduced fatigue levels than those with less social support. This finding emphasizes the importance of social support for HD patients by healthcare providers, health administrators, the community and family members to help them overcome their sleep and fatigue problems and improve their outcomes.

This study has several implications for nursing practice in Saudi Arabia in terms of assessing and treating fatigue and sleep problems in HD patients. Such implications may entail identifying the causes of fatigue, such as anemia and depression, and developing interventions to address them. Nurses should also be involved in educating HD patients on energy conservation methods and the need for physical activity. Interventions can be designed to address the underlying causes of poor sleep quality, such as pain and anxiety. Sleep hygiene and stress reduction are two other topics that nurses can discuss with patients. Nurses can help boost social support by collaborating with patients and family members, including their role in introducing patients to support groups or other services, as well as educating them and their families on the importance of social support. Nurses can assist in enhancing the QOL of Saudi Arabian HD patients by addressing social support, sleep disturbances, and fatigue.

## Data availability statement

The raw data supporting the conclusions of this article will be made available by the authors, without undue reservation.

## Ethics statement

This study was approved by the Research Ethics Committees at the University of Hail, Hail Health Clusters and the General Directorate of Health Affairs in Al-Qassim (Approval No.: H-2021-206, H120 08-L-074, and 607-44-2091, respectively). Anonymity and confidentiality were maintained during the study.

## Author contributions

BA: Conceptualization, Formal analysis, Methodology, Project administration, Supervision, Writing – original draft, Writing – review & editing. SA: Formal analysis, Methodology, Software, Writing – original draft, Writing – review & editing. EP: Writing – original draft, Writing – review & editing, Methodology. AA: Writing – review & editing. NM: Writing – review & editing. JE: Conceptualization, Methodology, Writing – review & editing. VB: Writing – review & editing, Formal analysis, Methodology, Validation. MA: Writing – review & editing. AR: Writing – review & editing. FA: Writing – review & editing.
